# Aging-Associated Augmentation of Gut Microbiome Virulence Capability Drives Sepsis Severity

**DOI:** 10.1128/mbio.00052-23

**Published:** 2023-04-27

**Authors:** James F. Colbert, Joshua M. Kirsch, Christopher L. Erzen, Christophe J. Langouët-Astrié, Grace E. Thompson, Sarah A. McMurtry, Jennifer M. Kofonow, Charles E. Robertson, Elizabeth J. Kovacs, Ryan C. Sullivan, Joseph A. Hippensteel, Namrata V. Sawant, Nicole J. De Nisco, Bruce D. McCollister, Robert S. Schwartz, Alexander R. Horswill, Daniel N. Frank, Breck A. Duerkop, Eric P. Schmidt

**Affiliations:** a Department of Medicine, University of Colorado School of Medicine, Aurora, Colorado, USA; b Department of Immunology and Microbiology, University of Colorado School of Medicine, Aurora, Colorado, USA; c Rocky Mountain Regional Veterans Affairs Medical Center, Aurora, Colorado, USA; d University of Colorado, Boulder, Colorado, USA; e Department of Surgery, University of Colorado School of Medicine, Aurora, Colorado, USA; f Department of Biological Sciences, University of Texas at Dallas, Richardson, Texas, USA; g Department of Urology, University of Texas Southwestern Medical Center, Dallas, Texas, USA; h Department of Medicine, Massachusetts General Hospital, Boston, Massachusetts, USA; Washington University School of Medicine in St. Louis; Universite de Geneve

**Keywords:** aging, host-pathogen interaction, metagenomics, microbiome, sepsis

## Abstract

Prior research has focused on host factors as mediators of exaggerated sepsis-associated morbidity and mortality in older adults. This focus on the host, however, has failed to identify therapies that improve sepsis outcomes in the elderly. We hypothesized that the increased susceptibility of the aging population to sepsis is not only a function of the host but also reflects longevity-associated changes in the virulence of gut pathobionts. We utilized two complementary models of gut microbiota-induced experimental sepsis to establish the aged gut microbiome as a key pathophysiologic driver of heightened disease severity. Further murine and human investigations into these polymicrobial bacterial communities demonstrated that age was associated with only subtle shifts in ecological composition but also an overabundance of genomic virulence factors that have functional consequence on host immune evasion.

## INTRODUCTION

Sepsis is a common, lethal, and incompletely understood clinical entity that disproportionally impacts the aging population (1, 2). Multiple epidemiologic studies have demonstrated a strong association of longevity with both sepsis incidence and case fatality rate (3, 4). Older patients are particularly likely to develop sepsis caused by bacteria originating in the gut microbiota via clinical syndromes such as bowel perforation, urinary tract infection, and aspiration pneumonia (5–7). As the worldwide population ages, there is increasing need to establish a mechanistic understanding of the sepsis-aging paradigm, allowing for the development of innovative therapeutic strategies personalized to the older population.

In the most simplistic terms, sepsis is a severe host-pathogen interaction. However, sepsis definitions (and research investigations) are largely focused on the host response to the pathogen as opposed to the pathogen itself (8). Within this framework, the pathogen is often seen as a static, homogeneous infectious insult that triggers the dysregulated host response. Accordingly, exaggerated sepsis severity outcomes in the aging population have been attributed to either an age-associated waning of immune function (i.e., immunosenescence) or an alteration in baseline inflammatory response (i.e., inflammaging) (9, 10). However, therapeutics targeting the host immune response or inflammatory cascade have consistently failed to improve clinical outcomes of septic patients in any age group (11, 12). Conversely, therapeutic strategies targeting the pathogen with antimicrobial agents have consistently demonstrated significant decreases in sepsis-associated morbidity and mortality (13, 14).

Given the importance of the pathogen to sepsis outcomes, we sought to determine if longevity-associated changes in gut microbial virulence contribute to aging-associated sepsis severity. We hypothesized that throughout the lifetime of the host, the gut microbiota becomes enriched with taxa and virulence factors that promote host immune evasion. Escape of these age-conditioned pathogens from the intestinal lumen therefore leads to exaggerated sepsis severity. This novel concept, that the gut microbiota also “ages” throughout the life span of the host and selects for hypervirulent pathobionts, has the potential to inform targeted therapeutic approaches to mitigate the burden of sepsis in older adults (3, 9, 15).

## RESULTS

### Gut microbiota-induced sepsis severity is determined by the age of the animal from which the infectious insult was derived.

**FIG 1:**
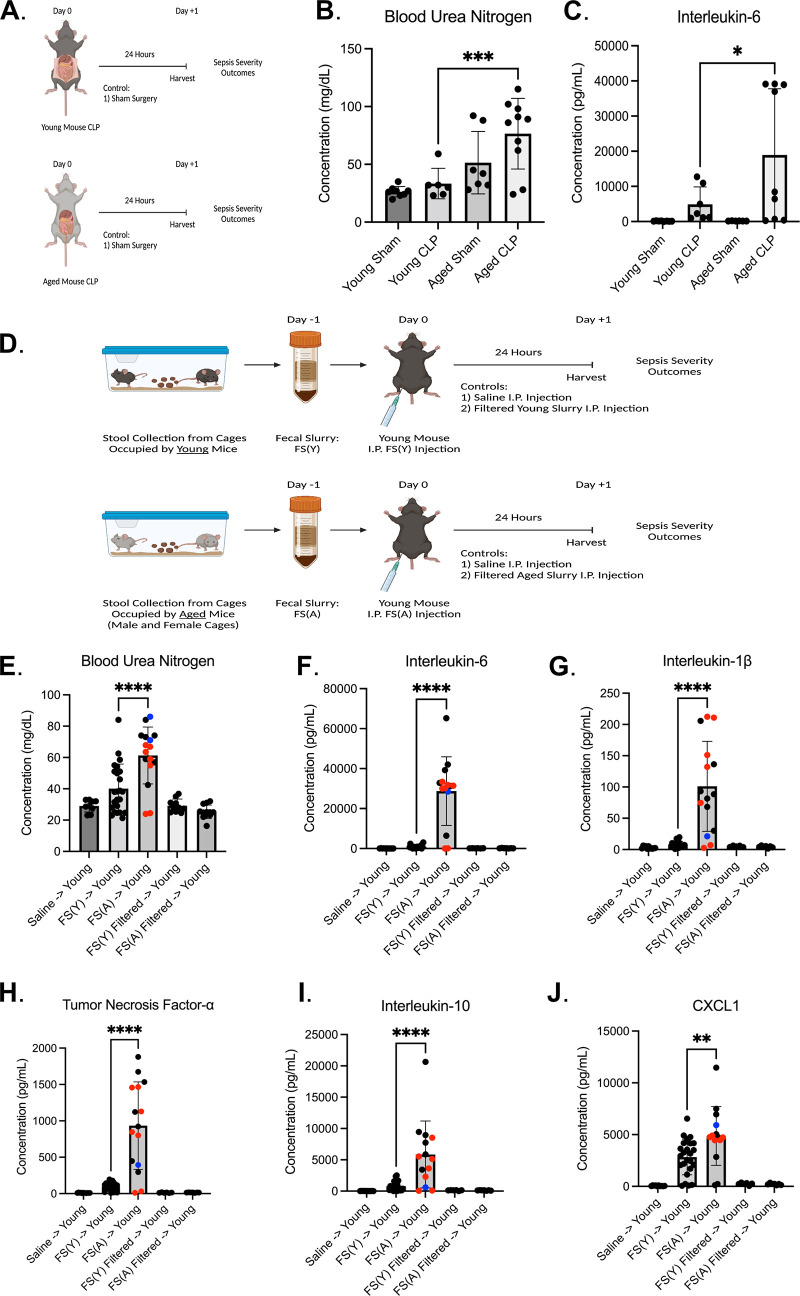
Age of the animal from which infectious insult is derived determines sepsis outcomes. (A to C) Experimental design and measurements of sepsis severity via blood urea nitrogen (B) and circulating interleukin-6 (C) after cecal ligation and puncture compared between young and aged mice and with contemporaneous sham surgery control. *n* = 7 to 12 animals per group. Experimental dropout (i.e., mortality) 0% in all groups besides aged CLP with 17% (2/12 animals) 24-h mortality. (D) The complementary fecal slurry experimental sepsis model was utilized to assess the relative contribution of the aged gut microbiota to sepsis severity phenotype in young mice. Sepsis severity markers include acute kidney injury (E), circulating plasma cytokines (F to I), and chemokines (J) 24 h after intraperitoneal fecal slurry injection. Intraperitoneal injection of saline and filtered slurry (0.22-μm filter) served as control conditions. *n* = 10 to 25 animals per group. Experimental dropout was 0% in all groups besides aged slurry [FS(A)] injection into young mice (32% mortality [8/25 animals]). Sex and origin of donor animals for FS(A) are noted as follows: black dots, aged male from the National Institute on Aging; blue dots, aged male from Denver, Colorado; and red dots, aged female from the National Institute on Aging. Pairwise statistical testing presented for experimental sepsis groups of interest only (young CLP group versus aged CLP group in panels B and C. FS(Y) injection into young mice versus FS(A) injection into young mice in panels E-J). *, *P < *0.05; **, *P < *0.01; ***, *P < *0.001; ****, *P *< 0.0001. Schematics (panels A and D) created with BioRender.com.

To determine the relative contribution of longevity-associated changes in the gut microbiome to sepsis severity, we utilized two complementary experimental sepsis models. Cecal ligation and puncture (CLP), the gold-standard model of experimental sepsis, induces physiologic sepsis via leakage of the animal’s own gut bacteria into the peritoneal space (16, 17). Although it was not the primary goal of our study, we employed CLP to demonstrate divergent sepsis severity outcomes in young mice and aged mice at the 24-h time point (Fig. 1A) and establish experimental relevance to sepsis outcomes in older humans. Similar to previously published reports (18, 19), aged mice exhibit increased mortality (17% [2/12] versus 0% in sham control and young mouse CLP), worsened acute kidney injury (Fig. 1B), and elevated plasma levels of the inflammatory cytokine interleukin-6 (Fig. 1C) after CLP (additional plasma cytokine data are presented in Fig. S1 in the supplemental material). To assess the attributable role of the infectious insult to this phenotype, we leveraged a second model of gut microbiota-induced experimental sepsis, intraperitoneal fecal slurry injection. In this model, cage stool is collected and suspended in sterile saline prior to centrifugation and injection (20). Importantly, the fecal slurry model allows us to directly compare the virulence of fecal microbiota collected from one mouse population {i.e., fecal slurry from cages containing young animals [“FS(Y)”]} versus fecal microbiota collected from another mouse population {i.e., fecal slurry from cages containing aged animals [“FS(A)”]}.

10.1128/mbio.00052-23.1 FIG S1 Plasma cytokine levels after CLP in young and aged mice. Circulating plasma cytokine concentrations for interleukin-1β (A), tumor necrosis factor-α (B), and interleukin-10 (C) 24 h after cecal ligation and puncture compared between young and aged mice and with contemporaneous sham surgery control. *n* = 7 to 12 animals per group. Experimental dropout (i.e., mortality), 0% in all groups besides aged CLP with 17% (2/12 animals) 24-h mortality. The analysis did not demonstrate statistical significance between experimental sepsis groups of interest (young CLP versus aged CLP). Download FIG S1, TIF file, 0.3 MB. Copyright © 2023 Colbert et al. 2023 Colbert et al. https://creativecommons.org/licenses/by/4.0/ This content is distributed under the terms of the Creative Commons Attribution 4.0 International license.

This novel experimental design allowed for characterization of the gut microbiota as a contributor to aging-associated augmentation of sepsis severity. Our primary model kept the age of the recipient animal constant (young) while altering the donor age of the infectious insult [FS(Y) versus FS(A)] (Fig. 1D). Intriguingly, we found that the exaggerated sepsis severity observed in aged mice undergoing CLP could be replicated in young mice by inducing experimental sepsis with aged microbiota [FS(A)] as measured by 24-h mortality [32% (8/25) versus 0% in FS(Y) and control groups], septic kidney injury (Fig. 1E), and plasma cytokine/chemokine response (Fig. 1F to J). FS(A) induced the same augmented sepsis severity when administered to aged mice, with 100% 24-h mortality (4/4) after injection with FS(A) and 0% 24-h mortality (0/4) post-FS(Y) injection. To determine if live bacteria were responsible for fecal slurry-induced sepsis, we filtered slurry through a 0.22-μm filter prior to injection. Mice receiving filtered slurry [either FS(Y) or FS(A)] experienced no mortality at 24 h, no kidney dysfunction, and no elevation of plasma cytokine levels compared to saline control animals (Fig. 1E to J).

To confirm that our findings were not simply an artifact of a unique batch of mice, we repeated these fecal slurry experiments using aged animals from multiple different sources, including the National Institute on Aging [black dots in FS(A), young group] and animals aged at different institutions in Colorado, United States (Denver Health Medical Center) [blue dots in FS(A), young group]. Similar findings were observed when fecal slurry was created from cages containing aged female mice [Fig. 1D to J, red dots in FS(A), young group], suggesting that the increased virulence of FS(A) was not influenced by biological sex.

### Longevity is not associated with an overabundance of known pathogenic taxa in the murine gut microbiome.

**FIG 2:**
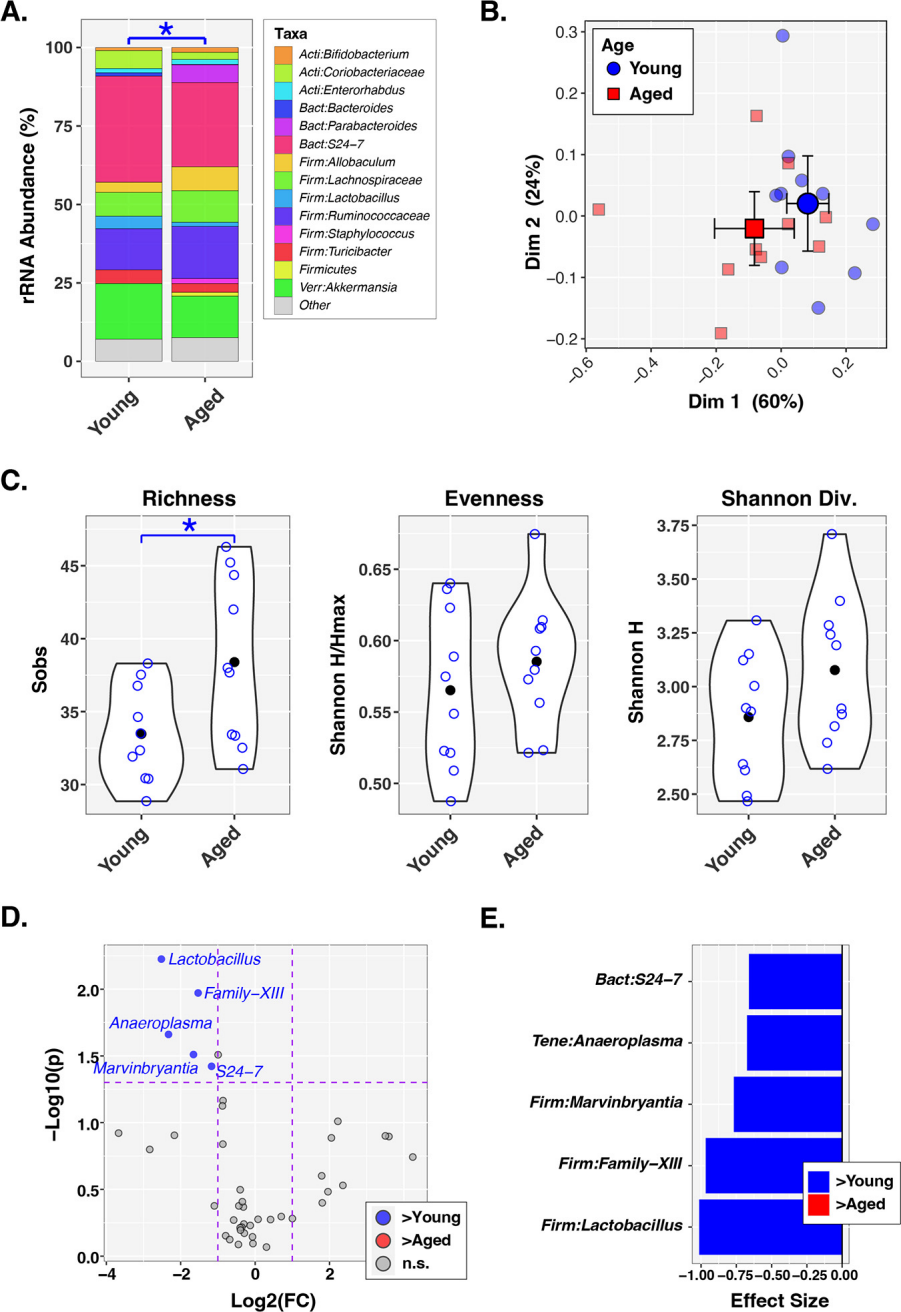
Longevity is not associated with an overabundance of known pathogenic taxa. Fecal slurry utilized for *in vivo* experiments subsequently underwent 16S rRNA gene sequencing and analysis for description of bacterial communities. *n *= 10 contemporaneous pairs of FS(Y) and FS(A), which are created from cages containing 5 animals per cage. (A) Relative abundance chart summarizing microbiota distributions between age groups. *, *P *< 0.05 measured by a permutational ANOVA (PERMANOVA) test with the Morisita-Horn dissimilarity index. PERMANOVA *P* values were inferred through 10^6^ label permutations. (B) Principal-coordinate analysis (PCoA) plot generated using the Morisita-Horn dissimilarity index. Individual slurry samples are shown as small symbols (circles, squares), while group mean PC1 and PC2 scores are indicated by larger symbols (circles, squares) with 95% confidence intervals. (C) Alpha diversity indices represented by violin plots indicating mean values (closed circles), individual data points (open circles), and overall distribution of values. *, *P < *0.05 as measured by ANOVA. (D) Volcano plot demonstrating differentially abundant taxa in young (blue) and aged (red) mice, as determined by ANOVA-like differential expression (ALDEx2) analysis with cutoffs of *P* value of <0.05 and log fold change of >2.0. (E) Effect sizes for individual significant taxa generated through ALDEx2 analysis.

Our phenotypic data led us to determine if there were differences in the overall composition of young versus aged murine gut bacterial communities. Utilizing 16S rRNA gene sequencing and analysis, we profiled microbiota present in the fecal slurry created from the two age groups. Beta diversity measures, as represented by relative abundance (Fig. 2A) and principal-component analysis (Fig. 2B), demonstrate an age-associated separation between groups (*P = *0.027), consistent with previously published reports (21–24). There was a statistically significant increase in richness of aged stool slurry (*P = *0.030) but no difference in evenness and overall alpha diversity (Shannon H index) (Fig. 2C). Only 5 genus-level taxa were more abundant in young stool slurry than aged (with a fold change of >2.0 and *P < *0.05); conversely, there were no overabundant taxa identified in aged stool slurry (Fig. 2D and E). Taken together, these data suggest that while there are expected differences in microbiota associated with the aging process, the increased virulence of FS(A) was not simply due to an overabundance of known pathogens.

### Aging is associated with an overabundance of bacterial virulence factors present in the murine gut microbiome.

**FIG 3:**
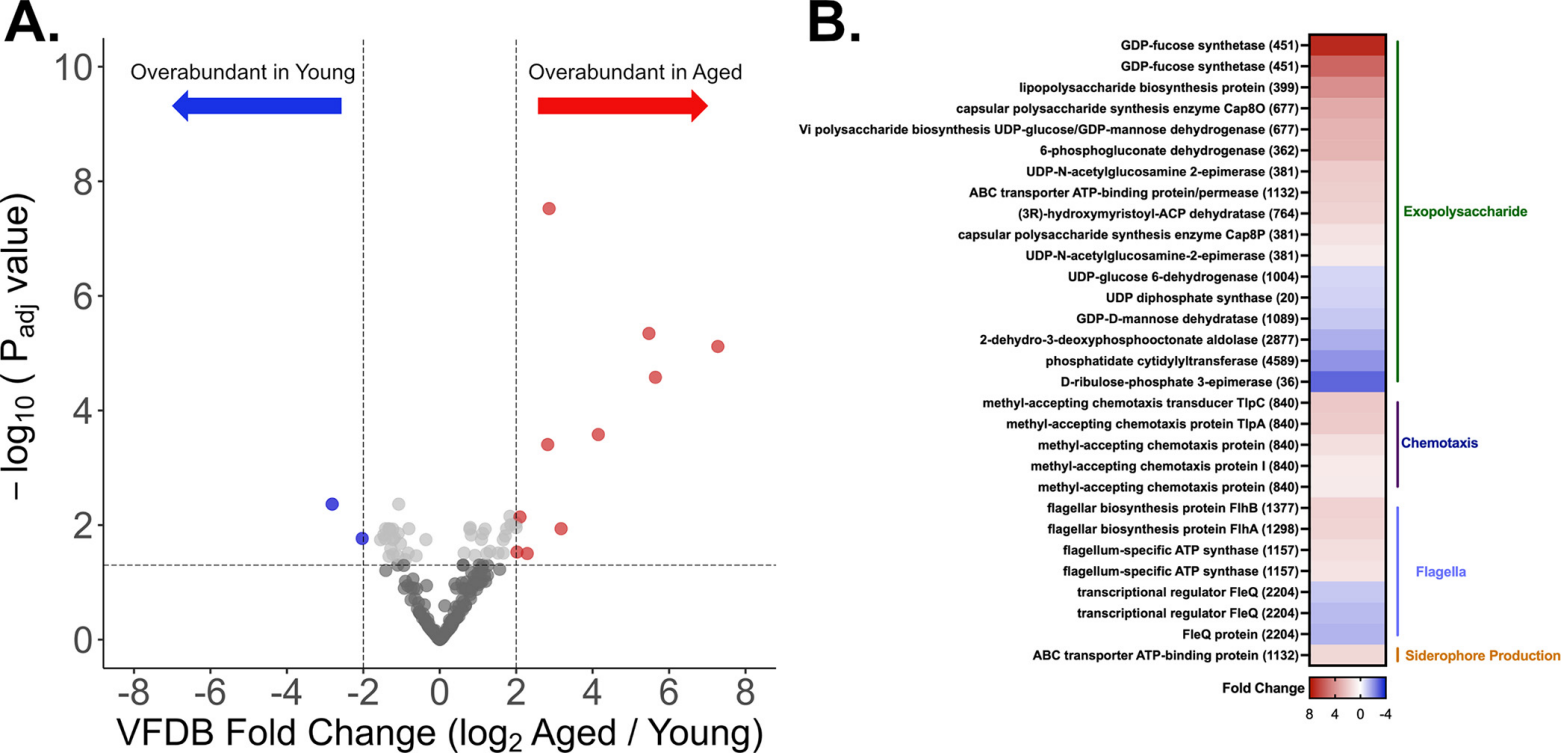
Aging is associated with an overabundance of virulence factors in the gut murine microbiome. The same fecal slurry samples with high-quality bacterial DNA [FS(Y), *n *= 8, and FS(A), *n *= 6] from *in vivo* experiments and 16S rRNA analysis underwent whole-genome shotgun sequencing and targeted metagenomic analysis focused on virulence factor abundance utilizing the Virulence Factor Database (VFDB) (25). (A) Volcano plot showing VFDB hits overabundant in FS(A) (red circles) versus FS(Y) (blue circles). Cutoff values of *P* of <0.05 and log-fold change >2. (B) Heatmap with all statistically significant (*P *< 0.05) individual virulence factor genes and relative fold change coded by color.

The relatively subtle differences in microbial community composition observed in FS(Y) and FS(A) did not readily explain the divergent sepsis severity outcomes noted in our experimental sepsis models (Fig. 1). To further investigate these communities, we pursued shotgun metagenomic sequencing and analysis of the same fecal slurry samples used for *in vivo* experiments and 16S rRNA gene sequencing in order to determine differences in the overall virulence capability of the bacterial communities between age groups. We utilized the Virulence Factor Database (VFDB) (25) to quantify the number and category of bacterial DNA sequences aligned against known proteins associated with virulence present in each group. Fig. 3A presents these data in volcano plot format, with heatmap and specific genes (and associated Cluster of Orthologous Genes [COG] [26] annotation in parentheses) coded for general virulence category (Fig. 3B). These findings demonstrate that FS(A) was enriched in bacterial genomes carrying more predicted virulence factors than FS(Y). This longevity-associated increase in microbiota virulence genes was driven by overrepresentation of genes predicted to encode exopolysaccharide synthesis, chemotaxis, flagella biosynthesis, and siderophore production pathways (Fig. 3B).

### Aging is associated with selection for pathogens resistant to blood killing in the murine gut microbiome.

**FIG 4:**
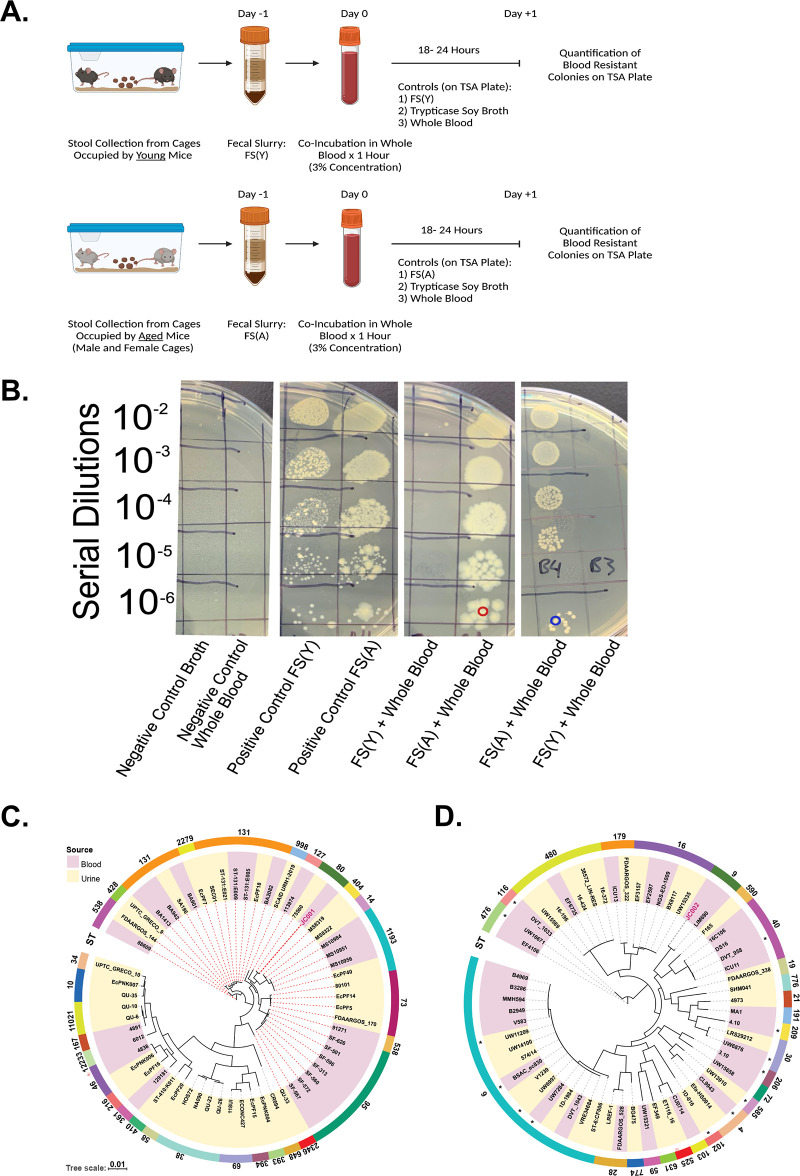
Aging selects for blood-resistant pathogens in the murine gut microbiome. (A) Study design demonstrating screening process for whole-blood killing resistance in fecal slurry. Biological replicates were performed using young mouse blood, and aged mouse blood (see Fig. S2 in the supplemental material). Control conditions included Trypticase soy broth (negative control), whole blood (negative control), and FS(Y) and FS(A) without coincubation with blood (positive control). After 1 h of coincubation in blood, samples were allowed to grow overnight followed by serial dilution and quantification of colonies. (B) Representative image showing all control conditions and two experimental replicates of FS(Y) plus whole blood and FS(A) plus whole blood. Blood-resistant colonies were identified by MALDI-TOF. (C, D) Red-circled colony, E. coli (JC001); blue-circled colony, E. faecalis (JC002), followed by whole-genome sequencing of isolates. Core genome phylogeny of E. coli and E. faecalis isolates from human blood and urine. Maximum-likelihood trees of publicly available E. coli (*n *= 59) and E. faecalis (*n *= 58) isolates as determined by Roary and RAxML along with JC001 and JC002 isolated in this study. Isolates are colored as per their source of origin indicated in the key (red, blood; yellow, urine). The phylogenetic tree was visualized and annotated using iTOL. ST denotes sequence type. An asterisk denotes that the ST was inconclusive, and the depicted ST is the nearest ST determined. E. coli clade B2 is indicated by red dotted lines. Schematic (panel A) created with BioRender.com.

To determine if this longevity-associated enrichment in microbiota virulence had functional consequence, we focused further investigation into genes identified in FS(A) compared to FS(Y), which may explain *in vivo* sepsis severity alterations. We found that the aging stool microbiome is enriched with an overabundance of virulence factors associated with potential host immune evasion strategies, including exopolysaccharide synthesis and maintenance (Fig. 3B). To determine the functional impact of these genetic differences on host immune evasion, we performed a proof-of-concept experiment and exposed FS(Y) and FS(A) to murine whole blood for 1 h to allow for host-mediated killing, followed by growth in media for 18 to 24 h prior to plating and quantification of surviving colonies (Fig. 4A). Of note, these experiments were performed in aerobic conditions and hence does not test for the possibility of host immunity-resistant obligate anaerobes. Strikingly, no FS(Y)-derived bacteria survived blood killing, while multiple phenotypic colonies from FS(A) survived at high quantities after exposure to murine whole blood (either from young mice or aged mice) (Fig. 4B; Fig. S2). Using matrix-assisted laser desorption ionization–time of flight mass spectrometry (MALDI-TOF MS), we identified that an Escherichia coli and an Enterococcus faecalis isolate [derived from FS(A)] survived whole-blood killing. Our findings suggest that the exaggerated sepsis severity noted in aged mice may be explained by an enhanced ability to avoid host immunity and is associated with an overabundance of predicted host immune resistance genes in the gut microbiota.

10.1128/mbio.00052-23.2 FIG S2 Donor age of whole blood does not impact pathogen blood resistance. (A) Similar to Fig. 4, study design demonstrating the screening process for whole-blood killing resistance in fecal slurry using aged mouse blood for the assay. Control conditions included Trypticase soy broth (negative control), whole blood (negative control), and FS(Y) and FS(A) without coincubation with blood (positive control). After 1 h of coincubation in blood, samples were allowed to grow overnight followed by serial dilution and quantification of colonies. (B) Representative image showing all control and experimental conditions. Schematic (panel A) created with BioRender.com. Download FIG S2, TIF file, 2.6 MB. Copyright © 2023 Colbert et al. 2023 Colbert et al. https://creativecommons.org/licenses/by/4.0/ This content is distributed under the terms of the Creative Commons Attribution 4.0 International license.

### Similar bacterial genetic features are seen in clinical bacterial isolates and the aged human gut microbiome.

To determine whether our murine findings are relevant to increased sepsis severity in aged humans, we performed an in-depth genetic analysis of our identified blood-resistant bacterial isolates by comparative genomics. E. coli and E. faecalis are both frequent human pathogens and known inhabitants of the human gut microbiota. We performed whole-genome sequencing of these bacterial survivors of whole blood (Fig. 4) and annotated their complete genomes (both chromosomal and nonchromosomal elements) (Data File S1) in reference to the VFDB (25). Fig. 4C and D presents phylogenetic trees of these organisms and their relatedness to human-derived clinical isolates from blood or urine samples (additional details regarding the clinical isolates are presented in Data File S2). The isolated E. coli belongs to phylogroup B2 (additional members of the phylogroup indicated with red dotted lines in Fig. 4C), which is associated with human disease and is closely related to multiple additional clinical isolates (27). The E. faecalis isolate was identified as sequence type 9, which has documented clinical relevance and relatedness to known clinically virulent strains (28).

10.1128/mbio.00052-23.5 DATA FILE S1 Virulence Factor Database annotation of complete blood-resistant E. coli (JC001) and E. faecalis (JC002) genomes, including COG number. Download DATA FILE S1, XLSX file, 0.02 MB. Copyright © 2023 Colbert et al. 2023 Colbert et al. https://creativecommons.org/licenses/by/4.0/ This content is distributed under the terms of the Creative Commons Attribution 4.0 International license.

10.1128/mbio.00052-23.6 DATA FILE S2 Comparison clinical E. coli and E. faecalis isolates utilized for comparative genomic and phylogenetic tree analyses. Download DATA FILE S2, XLSX file, 0.03 MB. Copyright © 2023 Colbert et al. 2023 Colbert et al. https://creativecommons.org/licenses/by/4.0/ This content is distributed under the terms of the Creative Commons Attribution 4.0 International license.

**FIG 5:**
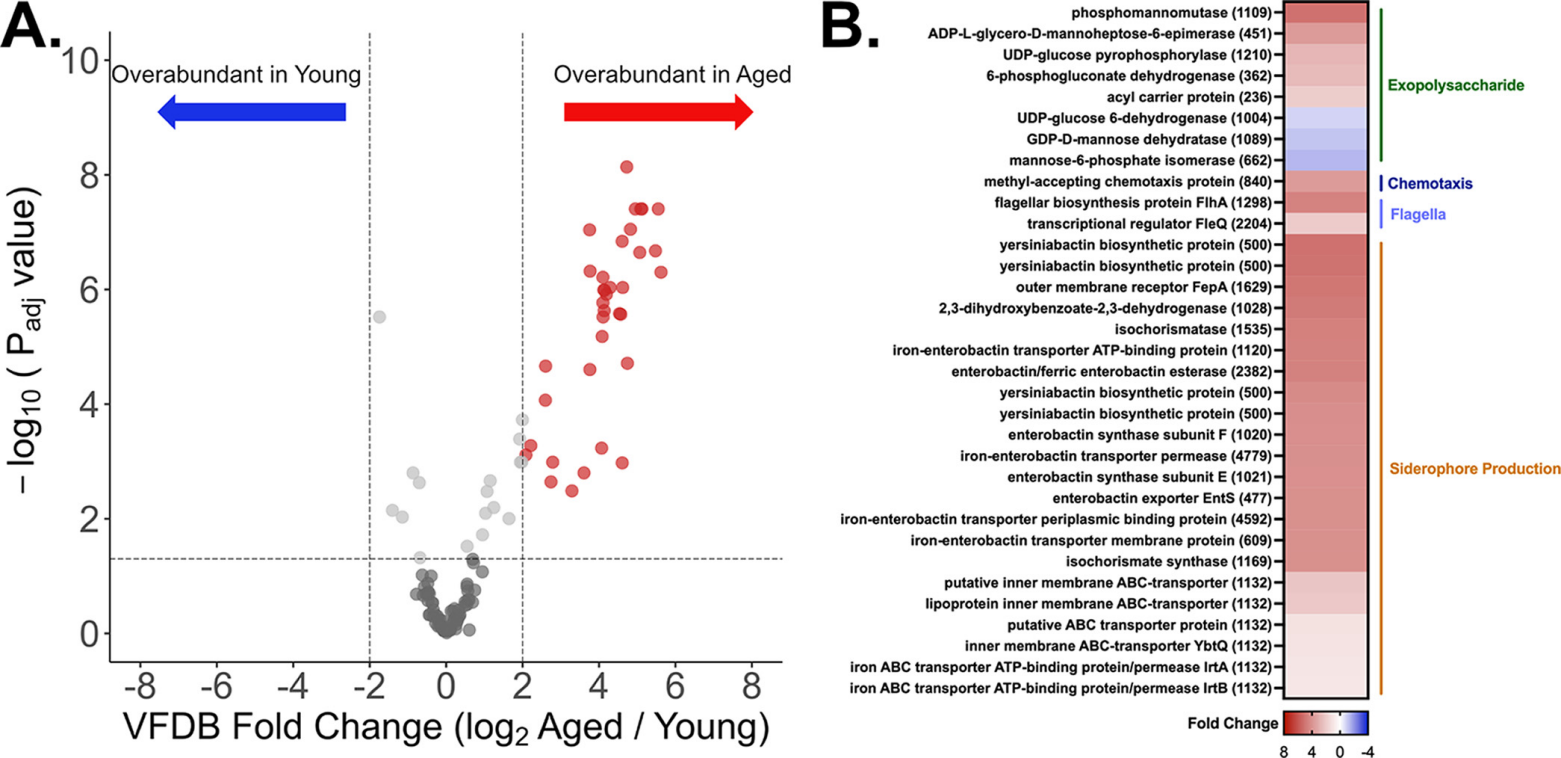
The aging human gut microbiome demonstrates an analogous overabundance of genomic virulence factors. Data from a previously published metagenomic data set (29) underwent the same analysis strategy as our murine data (Fig. 3). *n *= 62 total patients, *n *= 38 in the “aged human” group. (A) Volcano plot showing VFDB (25) hits overabundant in aged human gut microbiota (red circles) versus young human gut microbiota (blue circles). Cutoff values of *P* value of <0.05 and log fold change of >2. (B) Heatmap with all statistically significant (*P *< 0.05) individual virulence factor genes and relative fold change coded by color.

To further investigate the clinical relevance of our findings, we analyzed data from a previously published human gut metagenomic study to assess if there is a global shift toward aging-associated overabundance of virulence genes (29). Rampelli et al. generated fecal metagenomic data from 4 groups of patients with mean ages of 32.2, 72.5, 100.4, and 106.3 years, respectively. We combined the two oldest groups into one “aged human” group (*n *= 38 patients total) and performed the same analysis pathway from our murine studies on these data with the VFDB (25). The volcano plot and heatmap of individual genes (with COG annotation) demonstrate an overabundance of gut microbiome virulence genes associated with human aging (Fig. 5). Analogous to our studies of gut microbiome virulence associated with murine aging (Fig. 3), the overrepresented genes are classified as belonging to the same four mechanistic categories. The aged human microbiota was enriched in siderophore production genes, with a notable expansion of the number and fold change of yersiniabactin biosynthetic protein (COG0500) genes (Fig. S3) compared to aged murine counterparts.

10.1128/mbio.00052-23.3 FIG S3 Similar yersiniabactin operons are present in blood-resistant E. coli isolate and overabundant in the aged human gut microbiome. Comparison of yersiniabactin operons found in JC001 and aged human metagenomes. Genes labeled with lollipops and names were overabundant in aged human metagenomes. Color signifies similar gene function between operons. Coverage and identity between each aged human metagenome contig and the Ybt operon in JC001 were calculated with BLASTn. Download FIG S3, TIF file, 9.7 MB. Copyright © 2023 Colbert et al. 2023 Colbert et al. https://creativecommons.org/licenses/by/4.0/ This content is distributed under the terms of the Creative Commons Attribution 4.0 International license.

### Exopolysaccharide genes overabundant in the aging microbiome promote blood survival.

**FIG 6:**
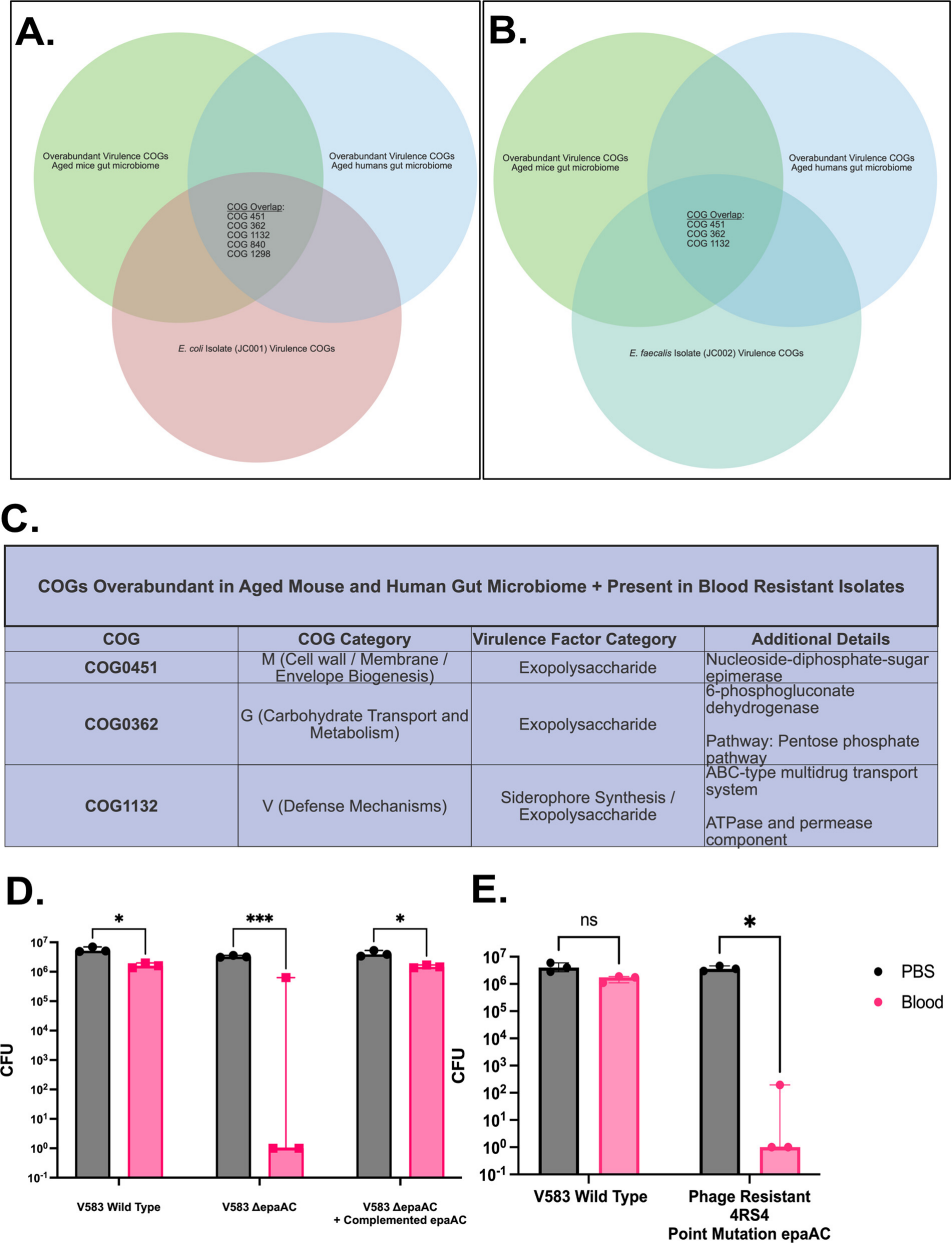
Identified exopolysaccharide virulence genes promote blood survival. (A) Venn diagram demonstrating Cluster of Orthologous Gene (COG) overlap between the aged murine gut microbiota, aged human gut microbiota, and isolated (blood-resistant) E. coli. (B) Analogous Venn diagram with isolated, blood-resistant E. faecalis. (C) Table highlighting in detail the three COGs overabundant in both murine and human gut microbiota, as well as both blood-resistant isolates. (D, E) Blood-killing assay of various strains of E. faecalis with genetic manipulation of the enterococcal polysaccharide antigen (*epaAC*) gene (COG0451). Data presented as percentage of PBS control (no blood killing). (D) Comparison of wild-type (V583 isolate) E. faecalis blood survival versus an engineered *epaAC* knockout and *epaAC* knockout plus plasmid complementation. (E) Comparison of V583 versus a phage-resistant strain (4RSR) with a missense point mutation in *epaAC* rendering the product nonfunctional. *n *= 9 per group (3 biological replicates and 3 technical replicates per group). Paired *t* tests with correction for multiple comparisons were applied to biological replicates (technical replicates were combined into one biological data point) with *, *P < *0.05; **, *P < *0.01; and ***, *P < *0.001.

One benefit of COG annotation is a deduplication of similar genes encoding similar proteins in various prokaryotic organisms. The COG database (NCBI) identifies 4,877 COGs associated with over 3 million protein IDs. Analysis of virulence genes present in the aged murine gut microbiota, aged human gut microbiota, blood-resistant E. coli, and blood-resistant E. faecalis demonstrated overlap of three specific COGs (COG0451, COG0362, COG1132) (Fig. 6A and B). All three of these COGs are associated with exopolysaccharide synthesis and could conceivably explain resistance to host killing via various mechanisms (Fig. 6C). As a proof-of-concept experiment, we genetically manipulated one of the identified overlapping COGs (COG0451) in a clinical strain of E. faecalis (V583, a human bloodstream isolate). The enterococcal polysaccharide antigen (Epa) operon has previously been described and noted to influence evasion of phagocytic killing (30), resistance to cationic antimicrobial peptides (31), and susceptibility to bacteriophage infection (32). We first utilized an engineered knockout of an Epa variable gene (*epaAC*, belonging to COG0451) in the V583 background. We conducted whole-blood killing experiments with wild-type V583, the V583 *epaAC* knockout strain (Δ*epaAC*), and a plasmid-complemented Δ*epaAC* strain to test the functional importance of this gene for blood survival. Figure 6D shows that the V583 Δ*epaAC* strain was more susceptible to blood killing than wild-type V583 and that resistance to blood killing could be restored by plasmid complementation of the *epaAC* gene. In addition to this engineered knockout strain, in a screen of bacteriophage-resistant E. faecalis (V583), we identified a naturally occurring strain (4RSR) with a missense point mutation in the *epaAC* gene, resulting in a V84L substitution. Similarly, this strain demonstrates markedly reduced blood survival, presumably due to a nonfunctional Epa product (Fig. 6E).

## DISCUSSION

Our findings highlight a previously unrecognized contributor to the pathophysiology of heightened aging-associated sepsis severity. To date, investigations of the intersection of aging and critical illness have focused on longevity-associated host processes such as waning immune function and alterations in inflammation. However, it is intuitive that the intestinal microbiota simultaneously undergoes genomic and phenotypic changes throughout the life span of the host organism. This aging of an enteric bacterial community likely selects for pathobionts with virulence factors that offer a fitness benefit, such as the evasion of host immunity, as demonstrated *ex vivo* by resistance to whole-blood killing (Fig. 4). Our work highlights that pathogen virulence factor genomics, and not simply the type of pathogen, is therefore a major mediator of mechanistic sepsis heterogeneity.

Importantly, this concept has the potential to inform the pursuit of novel biomarkers and therapeutic targets. The inherent complexity of these biological systems makes the discovery of a single unifying mechanism highly improbable; indeed, a single virulence factor is unlikely to fully explain the entirety of aging-associated sepsis severity. Our virulence-focused metagenomic analyses identified numerous overabundant age-associated virulence factors in the gut microbiota from both mice and humans. However, it is important to note that the individual genes were not identical between host species and that patient-level variability based on lifestyle is known to be greater in humans, which was not accounted for in our analyses. We pursued a mechanistic investigation into COG0451 due to the presence of this COG in blood-resistant isolates and both aged human and murine microbiota (Fig. 6), but there were additional examples of remarkable genomic overlap. Multiple siderophore biosynthetic genes were found to be overabundant in aged humans, and homologous biosynthetic clusters were found in the E. coli isolate (JC001) recovered from aged murine stool (see Fig. S3 in the supplemental material). One of these siderophore production operons produces yersiniabactin (Ybt), a siderophore implicated in the pathogenesis of multiple Gram-negative species (33–35). Ybt functionality increases E. coli virulence and has been shown to both increase availability of nutritional iron and protect against iron toxicity through sequestration mechanisms (35, 36). The operon encoding Ybt is contained in the *Yersinia* high-pathogenicity island, a mobilizable genomic component (37). The presence or absence of the various identified microbial genomic characteristics may provide the basis for accurate risk stratification and alternative antibacterial therapeutics designed to restore bacterial susceptibility to host immune factors.

The mechanisms underlying this microbial shift toward enhanced virulence capability with aging remain unexplored. We speculate that a slow evolutionary selective pressure associated with repeated host-pathogen interactions at the gut-mucosal barrier inform these changes in a stochastic fashion. However, the spread of mobilizable genomic features, such as the previously discussed *Yersinia* pathogenicity island in the aged microbiota, provides an alternative hypothesis for elevated longevity-associated virulence potential. It is also important to consider that this phenomenon may not be unique to aging; there may be analogous changes in other disease states, behaviors, or iatrogenic factors that facilitate exaggerated sepsis severity via a gut microbiota with an increased armament of virulence factors. This construct has the potential to better explain the heterogeneity of human sepsis outcomes based on underlying comorbidities, therapies, diet, and other factors, which requires further investigation.

There are more proximal potential clinical applications for the presented concept as well. A major clinical challenge is the prevention of secondary nosocomial infection in the intensive care unit, either post-primary infection, or post-noninfectious diagnosis. To address this concern, prior work has explored selective decontamination of the digestive tract with broad-spectrum enteral antibiotic therapy (38). While clinical benefit of this strategy has been demonstrated, it has not been widely adopted in the United States due to logistical hurdles and concern about the selection of multidrug-resistant organisms. This project lays the framework for sophisticated risk assessment of the virulence capacity of the gut microbiome and resulting targeted decontamination therapy to prevent nosocomial infection syndromes on an individual patient basis. Although speculative, our data suggest that directed bacteriophage therapy (as an alternative to broad-spectrum antibiotic therapy) may select for phage-resistant but as a trade-off—host-susceptible pathogens in the gut microbiota (Fig. 6).

Our work has additional, unanticipated implications for preclinical sepsis research and experimental sepsis models utilizing gut-derived bacteria (i.e., cecal ligation and puncture [CLP], the gold-standard model of experimental sepsis). In CLP, mice are exposed to their own gut microbiota. Our findings suggest that differences in CLP outcomes between different mouse genotypes may not merely be a function of host phenotype but also may reflect differential accumulation of virulence factors in enteric bacteria. It is thus plausible that microbial changes are driving outcomes in various murine models, including pretreated animals and transgenic strains. We encourage other researchers to take this variable into account in their mechanistic sepsis investigations, potentially by complementing CLP with fecal slurry modeling, using stool collected from a shared mouse donor.

Finally, our study creates a translational rationale supporting future prospective clinical studies. In our murine models, we have demonstrated an association of increased microbiota virulence with both age and with sepsis severity outcomes. While we note a similar increase in microbiome virulence and striking overlap of abundant virulence factors in the gut microbiota from older humans, additional prospective studies will be necessary to determine if this increase in bacterial virulence translates to worsened sepsis outcomes in aged humans. Another limitation of this work is a focus on bacterial aspects of the gut microbiome, although we acknowledge that fungal, archaeal, and viral components are also present in the mammalian gut microbiota. Due to a paucity of available techniques and lack of obvious aging-associated clinical relevance, we have not pursued investigations into these additional kingdoms of life in the current study.

In summary, our work identifies that aging-associated sepsis severity not only reflects host factors but also may be driven by the effects of longevity on pathogen virulence. These novel findings have major implications for our understanding of the mechanistic heterogeneity of critical illness, potentially guiding the personalization of sepsis care for the aged adult.

## MATERIALS AND METHODS

### Study design.

The overall objective of this research was to assess longevity-associated alterations in the gut microbiota and their relative contribution to sepsis severity. All animal experiments were conducted under approved the University of Colorado Institutional Animal Care and Use Committee (IACUC; protocol number 00307) and Institutional Biosafety Committee (IBC) protocols and in accordance with the Animal Research: Reporting of *In Vivo* Experiments (ARRIVE) guidelines (39). Individual animals were blindly randomized to experimental or control groups. Rigorous contemporaneous controls were used as described in individual experiments. For our primary experimental sepsis model (fecal slurry injection), we performed a power calculation, based on prior work, and estimated 10 to 12 animals per experimental group to detect a 50% difference in sepsis severity (continuous variables; acute kidney injury and plasma cytokine levels) with a power of 90% and α of 0.05. However, due to our higher than anticipated mortality [approximately one-third in FS(A)-injected animals], we doubled the number of animals in this experimental condition to account for dropout and to include biological sex [FS(A) created from aged female cages] as a variable. Statistical outliers were defined prior to experiments (GraphPad Prism ROUT method with recommended false-discovery rate [*q* value] of 1%) and were removed prior to analysis. Animal dropout (e.g., mortality) was recorded and explicitly reported. The intricacies of study design for specific experiments are presented in individual figures and figure legends.

### Animals.

Young male (8 to 10 weeks of age) C57BL/6 mice were obtained from Jackson Laboratory (Bar Harbor, ME) and allowed to acclimate in our local vivarium for 1 week prior to experimentation. As discussed in Results, we were concerned regarding the potential confounder of a “batch effect” of older animals, so we obtained aged animals (and stool) from various sources. Aged C57BL/6 male and female animals were acquired from the National Institute on Aging (Bethesda, MD) and allowed to acclimate for 1 week in Aurora, Colorado, prior to experimentation. Additionally, C57BL/6 mice born and aged in Denver, Colorado (at Denver Health Hospital, an academic partner of the University of Colorado) were utilized. Aged animals between the ages of 20 to 24 months were used for experiments. All animals were housed in the same vivarium room with identical diet, day/night cycle, and cleaning schedules.

### Experimental sepsis models.

As previously described, we have utilized two complementary experimental sepsis models to isolate the role of the gut microbiome as a driver of outcomes (20).

### (i) Cecal ligation and puncture.

Under inhaled isoflurane anesthesia, laparotomy was performed, followed by visualization and isolation of the cecum. The cecum was ligated with a 2-0 silk suture and punctured once through and through with a 23-gauge needle. The punctured cecum was returned to the abdominal cavity, and the incision was closed with 4-0 silk sutures. We then gave 500 μL of subcutaneous normal saline resuscitation. Analgesic subcutaneous buprenorphine and local bupivacaine were utilized. Sham surgery was identical to CLP, without ligation or puncture of the cecum (20).

### (ii) Fecal slurry injection.

Dry fecal pellets were collected 1 day before injection and suspended in sterile saline at a constant weight-to-volume ratio (180 mg stool per 1 mL of saline) and vortexed for 5 min. After storage overnight at 4°C, the sample was centrifuged (100 rpm × 5 min), and the liquid portion was removed. We injected 400 μL of fecal slurry via the intraperitoneal route to induce sepsis. We used 400 μL of sterile saline injection as a contemporaneous control. Fecal slurry was also filtered through a 0.22-μm filter to remove live bacteria and serve as an additional control (20).

### Sepsis severity outcomes.

**(i) Mortality.** Animals were observed after induction of sepsis by either model. Animals identified as being moribund were euthanized and recorded as mortality at 24 h.

**(ii) Acute kidney injury.** Plasma blood urea nitrogen (BUN), an indirect measurement of glomerular filtration rate, was quantified 24 h after sepsis induction using the QuantiChrom urea assay kit (BioAssay Systems, Hayward, CA). Working reagent from the kit was added to plasma samples, and optical density was read to 520 nm after 20 min of reaction time.

**(iii) Plasma cytokine and chemokine quantification.** Plasma interleukin-1β, interleukin-6, tumor necrosis factor α, interleukin-10, and CXCL1 were quantified using Meso Scale Diagnostics (Rockville, MD) custom multiplex electrochemiluminescence detection arrays.

### 16S rRNA microbiota analysis.

**(i) 16S rRNA amplicon library construction.** DNA was isolated from FS(Y) and FS(A) samples using the Qiagen PowerFecal DNA extraction kit. Bacterial profiles were determined by broad-range amplification and sequence analysis of 16S rRNA genes following our previously described methods (40, 41). In brief, amplicons were generated using primers targeting the V3-V4 variable region of the 16S rRNA gene. PCR products were normalized using a SequalPrep kit (Invitrogen, Carlsbad, CA), pooled, lyophilized, purified, and concentrated using a DNA clean and concentrator kit (Zymo, Irvine, CA). Pooled amplicons were quantified using Qubit fluorometer 2.0 (Invitrogen, Carlsbad, CA). The pool was diluted to 4 nM and denatured with 0.2 N NaOH at room temperature. The denatured DNA was diluted to 15 pM and spiked with 25% of the Illumina PhiX control DNA prior to loading the sequencer. Illumina paired-end sequencing was performed on the MiSeq platform with versions v2.4 of the MiSeq Control Software and MiSeq Reporter, using a 600-cycle version 3 reagent kit.

**(ii) Analysis of Illumina paired-end reads.** Illumina MiSeq paired-end reads were aligned to mouse reference genome mm10 with Bowtie 2, and matching sequences were discarded (42, 43). As previously described, the remaining nonmouse paired-end sequences were demultiplexed and then assembled using phrap (44, 45). Pairs that did not assemble were discarded. Assembled sequences were trimmed over a moving window of 5 nucleotides (nt) until average quality met or exceeded 20. Trimmed sequences with more than 1 ambiguity or shorter than 350 nt were discarded. Potential chimeras identified with Uchime (usearch6.0.203_i86linux32) (46) using the Schloss (47) Silva reference sequences were removed from subsequent analyses. Assembled sequences were aligned and classified with SINA (1.3.0-r23838) (48) using the 418,497 bacterial sequences in Silva 115NR99 (49) as a reference configured to yield the Silva taxonomy. Operational taxonomic units (OTUs) were produced by clustering sequences with identical taxonomic assignments. This process generated a median of 107,077 sequences per sample (interquartile range [IQR], 80,505 to 136,448). The software package Explicet (v2.10.5; http://www.explicet.org/) (50) was used to calculate alpha diversity scores through 1,000 replicate resamplings. All sequence libraries had goods coverage scores of ≥99%. All 16S rRNA gene sequence data and associated metadata were deposited in NIH GenBank’s Sequence Read Archive (SRA) under BioProject accession number PRJNA838636.

### Metagenomic analysis.

**(i) Bacterial DNA reads.** DNA was isolated from FS(Y) and FS(A) samples using the Qiagen PowerFecal DNA extraction kit, and whole-genome shotgun sequencing was performed by Novogene (Sacramento, CA). Human reads were obtained from SRA under BioProject accession number PRJNA553191 (29). Read decontamination and trimming were performed as previously described (51). In short, reads were trimmed using bbduk, part of the BBTools v38.90 bioinformatics tools using the following parameters: ktrim=l ktrim=r k=20 mink=4 minlen=20 qtrim=f ftl=20. Trimmed reads were mapped against human (hg38), mouse (mm39), and phiX174 genomes. Reads that did not map were used for the downstream analyses.

**(ii) Mapping to the Virulence Factor Database.** The core protein Virulence Factor Database (VFDB) (25) used in this study was downloaded on 28 June 2021. Decontaminated and trimmed reads were mapped against the database from both human and mouse samples using PALADIN v1.4.6 (52) with default settings. Total reads mapped to each virulence factor open reading frame were calculated using SAMtools v1.13 (53). Raw counts were used as input for DESeq2 v1.30.1 (54) to test for differential abundance, with zero-heavy rows eliminated from the count matrix before running the DESeq2 pipeline. All metagenomic gene sequence data and associated metadata were deposited in NIH GenBank’s SRA under BioProject accession number PRJNA844935.

### Whole-blood killing assay.

Murine whole blood was collected on the day of experiment via inferior vena cava collection. For fecal slurry experiments (Fig. 4), slurry was created as previously described and coincubated in whole blood at a concentration of 3% for 1 h at 37°C. After incubation, 100 μL of Trypticase soy broth was added to each well, and the samples were allowed to grow overnight at 37°C. After 18 to 24 h of growth, serial dilution was performed for quantification of blood-resistant colonies. For individual E. faecalis bacterial isolates (Fig. 6), the strains were incubated in whole human blood (collected under Colorado Multiple Institutional Review Board protocol 17-1926). Blood killing assays were performed on E. faecalis V583 and isogenic derivatives of V583, including BDU62 (Δ*epaAC*, EF2165), BDU62 pPL05 (*epaAC* complement), and 4RS4 (*epaAC* Val 84 Leu mutant). E. faecalis V583 and its *epaAC* derivatives have been described previously (32, 55). To determine the susceptibility of these strains to blood killing, each strain was grown overnight in brain heart infusion (BHI) broth at 37°C with shaking, and approximately 1 × 10^6^ CFU of each strain was added to either 90 μL of fresh human blood or phosphate-buffered saline (PBS), mixed, and incubated statically at 37°C for 1 h. We added 100 μL of BHI, and the samples were incubated at 37°C overnight. Samples were serially diluted and plated on BHI agar, and colonies were enumerated. Percent survival was calculated as the percentage of cells surviving blood killing compared to control cells in PBS.

### Bacterial identification by MALDI-TOF MS.

Fresh bacterial colony growth was deposited on a polished steel MSP 96 target (Bruker Daltonics, Leipzig, Germany) and covered with 1 μL of a 70% formic acid solution. Following air drying, the bacterial spot was overlaid with 1 μL of a saturated α-cyano-4-hydroxycinnamic acid (HCCA) matrix solution (Bruker Daltonics). Mass spectra were acquired and analyzed using a microflex LT mass spectrometer (Bruker Daltonics) in combination with research-use-only (RUO) versions of the MALDI Biotyper software (MBT Compass v4.1) and the reference database v9.0.0.0 (8,468 spectra covering 2,969 species). Calibration was done by following the manufacturer's instructions and using the manufacturer's recommended bacterial test standard. Bacterial species were assigned for scores of ≥2.0.

### Comparative genomics.

**(i) Sequencing and assembly.** The genomes of isolates JC001 and JC002 underwent whole-genome sequencing and assembly by the Microbial Genome Sequencing Center (MiGS, Pittsburgh, PA). Genomic sequence data were deposited in NIH GenBank’s SRA under BioProject accession number PRJNA844935 with individual accession numbers (JC001, BioSample accession number SAMN28860864; JC002, BioSample accession number SAMN28860875).

**(ii) Genome annotation.** Open reading frames were predicted and annotated using RASTtk (56). Contigs were assembled using megahit v1.2.7 with the “meta-large” preset (57) and open reading frames predicted and annotated using RASTtk with the determined annotations from the blood-killing isolates against metagenome contigs using BLASTn (58). Alignments between contigs were also performed using pairwise BLASTn. Contig visualization was performed using the gggenes package (v0.4.1) in R (v4.0.5).

**(iii) Pan-genome analysis.** Isolates JC001 and JC002 were analyzed with reference to publicly available genomic data of 59 human E. coli (blood, *n* = 24; urine, *n* = 35) and 58 human E. faecalis (blood, *n* = 29; urine, *n* = 29) isolates collected from the NCBI genome database. Multilocus sequence typing (MLST) (59) was performed using the MLST web server (available at https://pubmlst.org/) and the “Escherichia coli #1” and “Enterococcus faecalis” configurations. Genomes of all 119 isolates were annotated using Prokka (60). Core genome alignments were generated using the Roary (61) pipeline and were then used to construct maximum-likelihood (ML) trees using RAxML (62). iTOL (63) was used to visualize the phylogenetic trees and metadata.

**(iv) E. coli phylotyping.** JC001 was determined to belong to E. coli phylogroup B2 by examining the genome for the presence of the *chuA*, *yjaA*, and the DNA fragment TSPE4.C2 and using the Clermont classification system (64).

### Statistical analysis.

For *in vivo* experiments, prior to analysis, statistical outliers were identified as prespecified using the ROUT method in GraphPad Prism, with a *q* value of 1%. Biological and technical replicates (*n*) and dropout (mortality) are noted in individual figure legends. Comparisons between multiple independent groups were conducted using an ordinary one-way analysis of variance (ANOVA). Statistical significance of comparisons of interest (noncontrol conditions) was performed via Sidak’s multiple-comparison tests. Whole-blood killing quantification statistical significance was assessed using paired *t* tests. A *P* value of <0.05 was considered statistically significant. For 16S rRNA gene sequence analysis, differences in overall composition (i.e., beta-diversity) were assessed through permutational ANOVA (PERMANOVA [65, 66]) with the Morisita-Horn dissimilarity index. PERMANOVA *P* values were inferred through 10^6^ label permutations. Principal-coordinate analysis (PCoA) was carried out using the vegan wcmdscale function with dissimilarities measured using the Morisita-Horn index. Alpha-diversity indices (i.e., *S*_obs_, Shannon H, Shannon H/Hmax) were assessed by ANOVA. Individual taxa differing between treatment groups were identified using the ANOVA-like differential expression (ALDEx2) R package (67, 68). Additional specifics of statistical strategy for genomic analyses are presented in individual subheadings above.

### Data availability.

All data are available in the main text or the supplemental material. Genomic data and associated metadata were deposited in NIH GenBank’s SRA under the following accession numbers: murine gut microbiome 16S rRNA, BioProject accession number PRJNA838636; murine gut microbiome metagenomic data, BioProject accession number PRJNA844935; individual bacterial isolates, BioProject accession number PRJNA844935; JC001 (E. coli), BioSample accession number SAMN28860864; and JC002 (E. faecalis), BioSample accession number SAMN28860875.

10.1128/mbio.00052-23.4 TABLE S1 Specific aging-associated differential virulence factors in the murine and human gut microbiome. VFDB hits from Fig. 3 and Fig. 5 are listed by species (mouse versus human), virulence factor category, and association with aging (red, overabundant in aged; blue, overabundant in young). Log fold change and *P* value are listed for each individual gene. Download Table S1, PDF file, 1.0 MB. Copyright © 2023 Colbert et al. 2023 Colbert et al. https://creativecommons.org/licenses/by/4.0/ This content is distributed under the terms of the Creative Commons Attribution 4.0 International license.
