# Supplementary figures and images for: Aging-Associated Augmentation of Gut Microbiome Virulence Capability Drives Sepsis Severity
Source: mBio. 2023 Apr 27;14(3):e00052-23. doi: 10.1128/mbio.00052-23 (PMC10294665; doi:10.1128/mbio.00052-23)

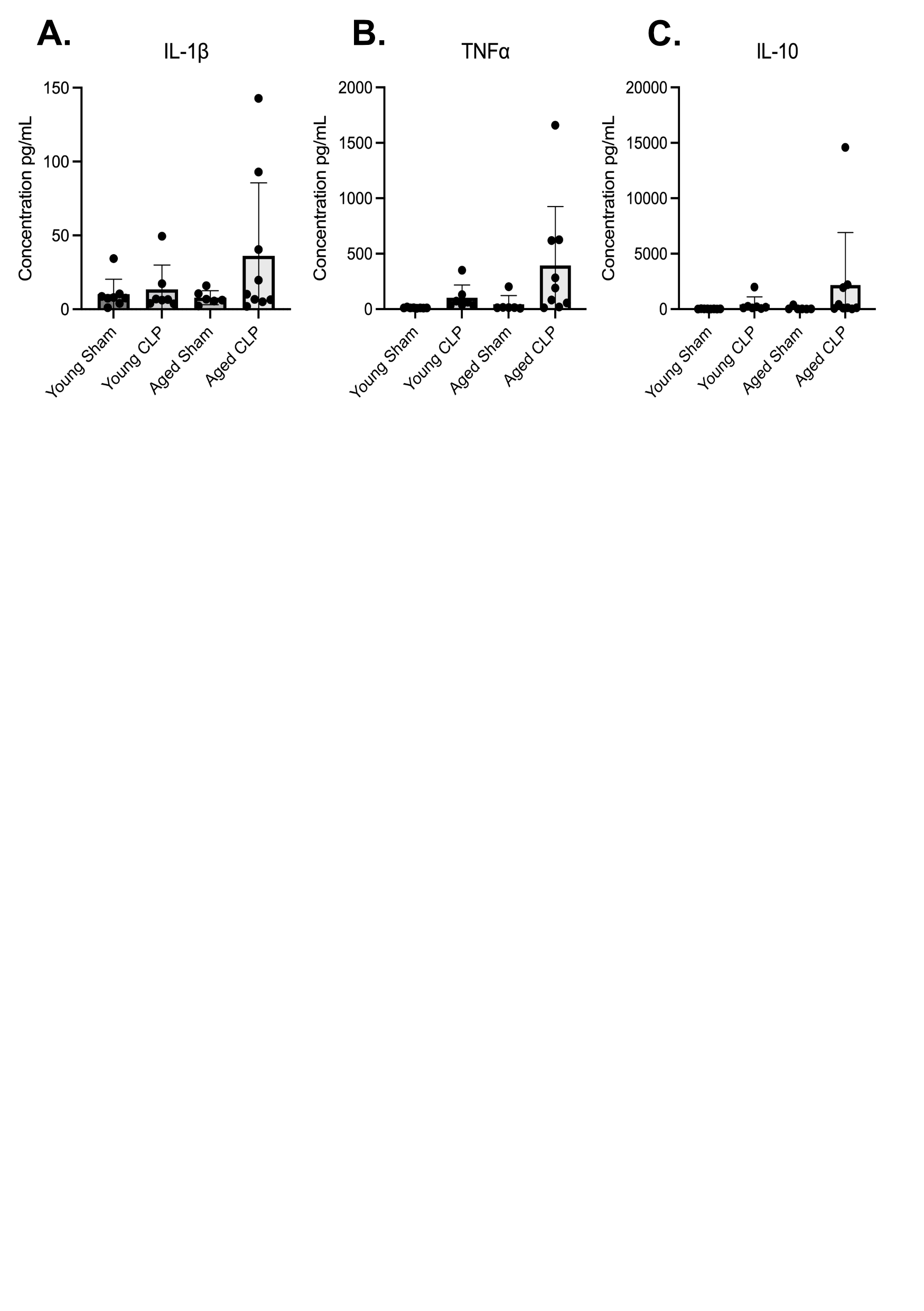

Supplement: FIG S1 [file mbio.00052-23-s0003.tif]

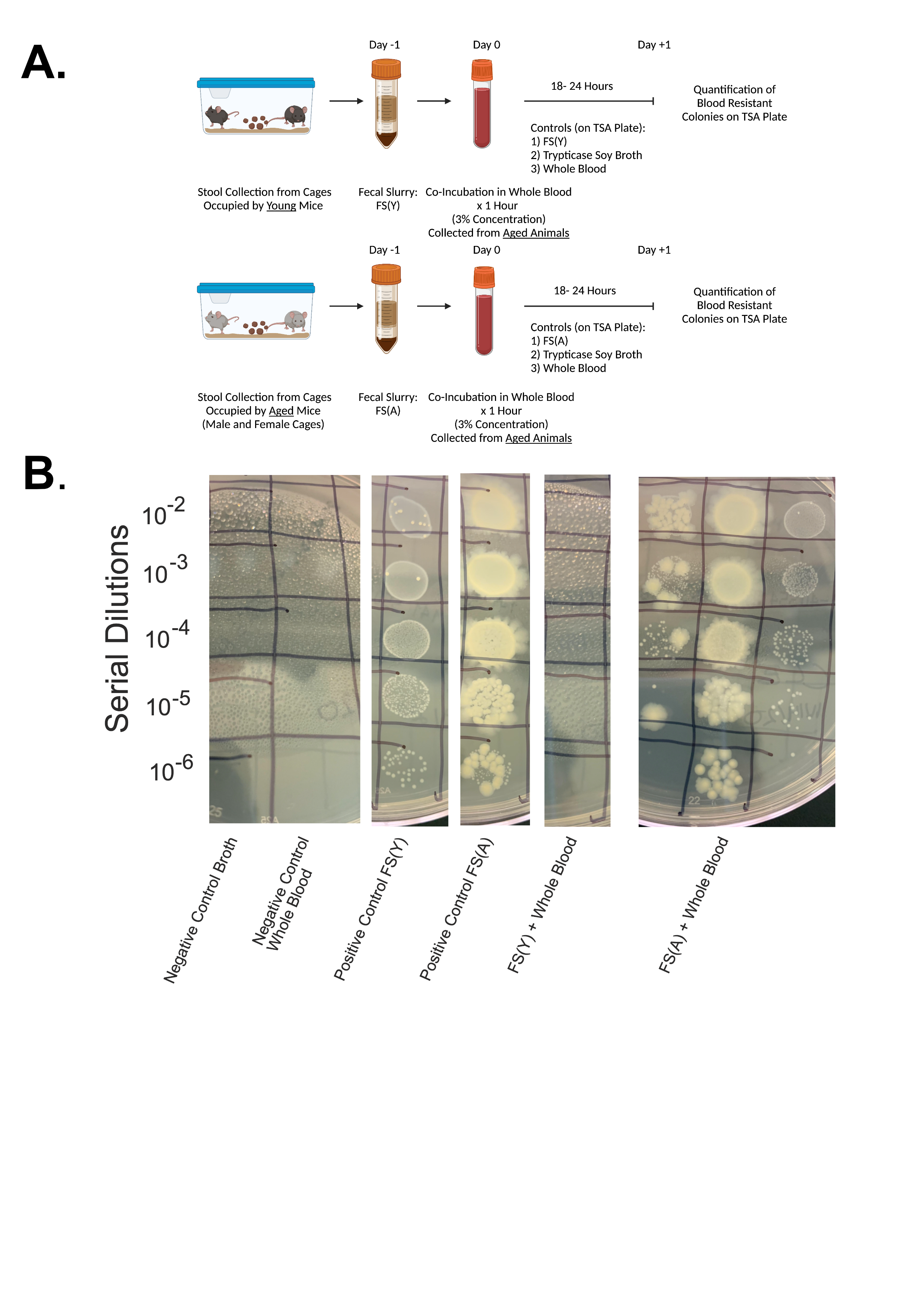

Supplement: FIG S2 [file mbio.00052-23-s0004.tif]

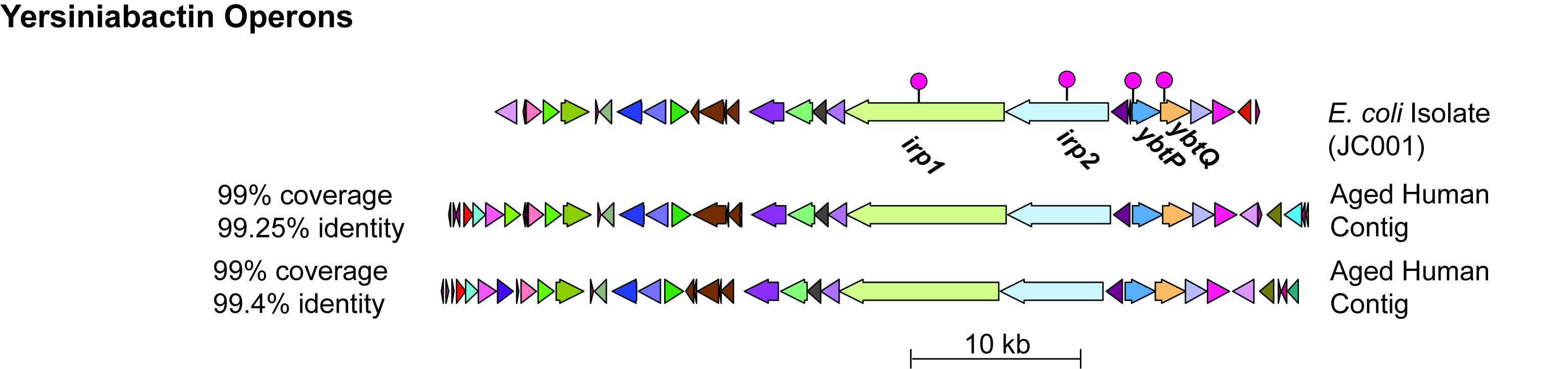

Supplement: FIG S3 [file mbio.00052-23-s0005.tif]
