# Supplementary material for: Aging-Associated Augmentation of Gut Microbiome Virulence Capability Drives Sepsis Severity
Source: mBio. 2023 Apr 27;14(3):e00052-23. doi: 10.1128/mbio.00052-23 (PMC10294665; doi:10.1128/mbio.00052-23)
Supplement: TABLE S1 [file mbio.00052-23-s0006.pdf]

Category	Mice DE VFs	Log2 Fold Change	*p* Value	Human DE VFs	Log2 Fold Change	*p* Value
**Exopolysaccharide**	GDP-fucose synthetase (451)	7.28	7.6E-06	Phosphomannomutase (1109)	5.12	3.9E-08
	GDP-fucose synthetase (451)	5.47	4.5E-06	ADP-L-glycero-D-mannoheptose-6-epimerase (451)	3.77	4.8E-07
	lipopolysaccharide biosynthesis protein (399)	4.15	2.6E-04	UDP-glucose pyrophosphorylase (1210)	2.79	1.0E-03
	capsular polysaccharide synthesis enzyme Cap8O (677)	3.17	1.2E-02	6-phosphogluconate dehydrogenase (362)	2.60	2.2E-05
	Vi polysaccharide biosynthesis UDP-glucose/GDP-mannose dehydrogenase (677)	2.86	3.0E-08	acyl carrier protein (236)	1.93	4.1E-04
	6-phosphogluconate dehydrogenase (362)	2.82	3.9E-04	UDP-glucose 6-dehydrogenase (1004)	-0.87	1.6E-03
	UDP-N-acetylglucosamine 2-epimerase (381)	2.00	9.3E-03	GDP-D-mannose dehydratase (1089)	-1.15	9.3E-03
	ABC transporter ATP-binding protein/permease (1132)	1.86	9.9E-03	mannose-6-phosphate isomerase (662)	-1.41	7.1E-03
	(3R)-hydroxymyristoyl-ACP dehydratase (764)	1.71	1.5E-02			
	capsular polysaccharide synthesis enzyme Cap8P (381)	1.12	1.4E-02			
	UDP-N-acetylglucosamine-2-epimerase (381)	0.79	1.2E-02			
	UDP-glucose 6-dehydrogenase (1004)	-0.83	3.1E-02			
	UDP diphosphate synthase (20)	-0.90	4.0E-02			
	GDP-D-mannose dehydratase (1089)	-1.09	1.4E-02			
	2-dehydro-3-deoxyphosphooctonate aldolase (2877)	-1.55	1.8E-02			
	phosphatidate cytidylyltransferase (4589)	-2.03	1.7E-02			
	D-ribulose-phosphate 3-epimerase (36)	-2.81	4.3E-03			
**Chemotaxis**	methyl-accepting chemotaxis transducer TlpC (840)	2.10	7.2E-03	methyl-accepting chemotaxis protein (840)	3.75	9.1E-08
	methyl-accepting chemotaxis protein TlpA (840)	2.00	1.1E-02			
	methyl-accepting chemotaxis protein (840)	1.23	3.1E-02			
	methyl-accepting chemotaxis protein I (840)	0.82	1.5E-02			
	methyl-accepting chemotaxis protein (840)	0.80	1.1E-02			
**Flagella**	flagellar biosynthesis protein FlhB (1377)	1.75	1.2E-02	flagellar biosynthesis protein FlhA (1298)	4.54	2.6E-06
	flagellar biosynthesis protein FlhA (1298)	1.66	1.8E-02	transcriptional regulator FleQ (2204)	1.98	1.0E-03
	flagellum-specific ATP synthase (1157)	1.31	2.9E-02			
	flagellum-specific ATP synthase (1157)	1.09	1.8E-02			
	transcriptional regulator FleQ (2204)	-1.10	3.5E-02			
	ranscriptional regulator FleQ (2204)	-1.32	1.2E-02			
	FleQ protein (2204)	-1.47	1.5E-02			
**Siderophore Production**	ABC transporter ATP-binding protein (1132)	1.53	3.1E-02	2,3-dihydroxybenzoate-2,3-dehydrogenase (1028)	4.82	8.9E-08
				outer membrane receptor FepA (1629)	4.95	3.9E-08
				Isochorismatase (1535)	4.62	9.2E-07
				iron-enterobactin transporter ATP-binding protein (1120)	4.61	1.4E-07
				enterobactin/ferric enterobactin esterase (2382)	4.57	2.7E-06
				enterobactin synthase subunit F (1020)	4.14	1.0E-06
				iron-enterobactin transporter permease (4779)	4.13	2.3E-06
				enterobactin synthase subunit E (1021)	4.13	1.0E-06
				enterobactin exporter EntS (477)	4.11	3.0E-06
				iron-enterobactin transporter periplasmic binding protein (4592)	4.10	1.7E-06
				iron-enterobactin transporter membrane protein (609)	4.10	6.1E-07
				isochorismate synthase (1169)	4.08	6.6E-06
				iron ABC transporter ATP-binding protein/permease IrtA (1132)	1.03	8.0E-03
				iron ABC transporter ATP-binding protein/permease IrtB (1132)	0.95	1.9E-02
				yersiniabactin biosynthetic protein (500)	5.09	3.9E-08
				yersiniabactin biosynthetic protein (500)	5.07	2.3E-07
				yersiniabactin biosynthetic protein (500)	4.29	9.2E-07
				yersiniabactin biosynthetic protein (500)	4.19	1.2E-06
				putative inner membrane ABC-transporter (1132)	2.21	5.3E-04
				lipoprotein inner membrane ABC-transporter (1132)	2.09	7.7E-04
				putative ABC transporter protein (1132)	1.15	2.2E-03
				inner membrane ABC-transporter YbtQ (1132)	1.07	3.3E-03

Table S1. Specific aging-associated differential virulence factors in the murine and human gut microbiome.

VFDB hits from Fig 3 and Fig 5 are listed by species (mouse vs human), virulence factor category, and association with aging (red = overabundant in aged, blue = overabundant in young). Log-fold change and *p* value are listed for each individual gene.
